# Surgical peritoneal dialysis catheter placement under local versus general anesthesia: impact on clinical safety, efficiency, and resource utilization

**DOI:** 10.3389/fmed.2026.1779652

**Published:** 2026-03-04

**Authors:** Martin Reichert, Sarah Mätzig, Faeq Husain-Syed, Daniel Strack, Michael Sander, Christian Koch, Andreas Hecker, Anca-Laura Amati

**Affiliations:** 1 Department of General, Visceral, Thoracic and Transplant Surgery, Giessen University Hospital, Justus-Liebig-University Giessen, Giessen, Germany; 2 Division of Nephrology, Department of Internal Medicine II, Giessen University Hospital, Justus-Liebig-University Giessen, Giessen, Germany; 3 Department of Anesthesiology, Intensive Care Medicine and Pain Therapy, Giessen University Hospital, Justus-Liebig-University Giessen, Giessen, Germany

**Keywords:** local anesthesia, perioperative medicine, peritoneal dialysis, resource utilization, surgical peritoneal dialysis catheter placement

## Abstract

**Introduction:**

Surgical peritoneal dialysis (PD) catheter implantation can be performed under various anesthetic strategies, evidence guiding the optimal approach regarding clinical safety and perioperative efficiency remains limited. This study evaluated perioperative outcomes and resource utilization in open surgical PD catheter implantation under general (GA) versus local/regional anesthesia (LA).

**Methods:**

This retrospective single-center cohort study included all open surgical PD catheter implantations performed between 2010 and 2021. Clinical outcomes and perioperative workflow parameters were analyzed. Patients were stratified by anesthesia type (GA versus LA), comorbidities, and operating room (OR) isolation status related to multidrug-resistant organisms.

**Results:**

A total of 508 procedures were included (419 GA, 89 LA). Patients undergoing LA were older and more comorbid, with 49.4% classified as ASA ≥ 4 versus 14.6% in the GA group. Surgical procedures were comparable. LA was associated with shorter OR and post-anesthesia care unit times and faster transfer to definitive care units, indicating more efficient perioperative management. Postoperative surgical complication rates were comparable. Prolonged intensive care treatment occurred more frequently in LA patients, likely reflecting higher baseline illness severity. In patients with ASA ≥ 4, LA showed a trend toward reduced intraoperative catecholamine use (47.7% versus 67.2%; *p* = 0.0697). In multivariable analyses adjusting for age, ASA score, and cardiopulmonary comorbidities, anesthetic strategy was not independently associated with major safety outcomes. Among patients requiring isolation, GA resulted in disproportionate OR occupancy, whereas LA facilitated more efficient workflow regardless of isolation status.

**Discussion:**

LA is preferentially used in high-risk patients. After adjustment for baseline risk, its surgical safety is comparable to GA, while offering perioperative resource and organizational advantages. Tailoring anesthetic strategies to patient comorbidities and isolation requirements may improve perioperative workflow and resource utilization without compromising outcomes.

## 1 Introduction

Peritoneal dialysis (PD) is an essential renal replacement therapy for patients with chronic kidney failure, offering advantages such as patient autonomy, preservation of residual renal function, and potentially improved quality of life in comparison with hemodialysis (1, 2). The success of PD therapy critically depends on reliable peritoneal access through properly functioning catheters, making the catheter insertion technique a pivotal factor in treatment outcomes (3).

Several surgical approaches for PD catheter implantation have been established, including percutaneous, laparoscopic, and open surgical techniques, each with specific advantages and limitations (4, 5). Among these, open implantation through mini-laparotomy remains a widely used technique (6). While this approach allows precise catheter placement, it is resource- and personnel-intensive, making it a crucial target for process optimization, especially in times of healthcare workforce shortages. Given the increasing burden on surgical and anesthesiology teams, strategies to streamline procedures without compromising patient safety and clinical outcomes are essential.

Despite the widespread use of various insertion techniques, evidence guiding optimal perioperative management remains limited. Recent Cochrane reviews have highlighted the heterogeneity in study designs and small sample sizes, which have resulted in insufficient evidence-based recommendations for clinicians regarding optimal PD catheter insertion protocols (4, 5). This evidence gap is particularly concerning given the increasing prevalence of kidney failure and the growing demand for home-based dialysis services (7, 8).

Selecting the most suitable anesthetic approach is a key factor in ensuring successful PD catheter placement and efficient perioperative management. Current options range from local infiltration anesthesia and regional blocks to general anesthesia (GA) (9–14). Although, GA provides optimal surgical conditions for intraabdominal procedures, it entails substantial perioperative risks, particularly in multimorbid patients with kidney failure (9, 12). These patients typically have elevated American Society of Anesthesiologists (ASA) scores and increased perioperative morbidity and mortality risks under GA (15). Local anesthesia (LA) techniques represent potentially safer alternatives in high-risk patients (9–14). Several studies have demonstrated the feasibility and safety of LA approaches for PD catheter insertion, with high success rates and minimal need for conversion to GA (9, 12–14).

Beyond clinical considerations, the choice of the anesthetic technique has major implications for healthcare resource utilization and economic efficiency. Operating rooms (OR) represent the highest revenue-generating areas in hospitals, with high contribution margins per hour, making operational efficiency critically important (16–18). The perioperative workflow, which includes the preoperative preparation time, anesthesia induction, surgery duration, and postoperative monitoring requirements, directly influences OR capacities and hospital economics (19–21). With healthcare systems facing increasing economic pressure and staff shortages, optimizing perioperative processes while maintaining patient safety has become paramount (18). Reducing unnecessary resource consumption while maintaining high-quality patient care is critical to ensure the sustainability of PD catheter insertion services.

An additional complexity in contemporary perioperative management involves patients colonized with multidrug-resistant (MDR) organisms, who require isolation protocols in the OR environment. These isolation requirements can substantially influence OR efficiency and resource allocation, particularly when extended postoperative monitoring is required (22, 23). The interaction between anesthetic choice and isolation requirements has not been systematically evaluated in the context of PD catheter placement.

Therefore, this study aimed to evaluate the influence of different anesthetic approaches during open surgical PD catheter implantation on perioperative efficiency, economic burden, and clinical outcomes. By analyzing a large retrospective cohort, we sought to identify the most effective perioperative management strategy that balances patient safety, procedural efficiency, and resource utilization particularly in high-risk patients and those requiring isolation precautions. The real-world data from this study will guide future perioperative management strategies to optimize perioperative efficiency without compromising patient safety.

## 2 Materials and methods

### 2.1 Patients

This retrospective single-center cohort study was formally approved by the local ethics committee of the medical faculty of the University of Giessen (approval No. 226/20). The study was performed in accordance with the latest version of the Declaration of Helsinki. All patients were treated according to the institutional standard of care.

**Figure 1:**
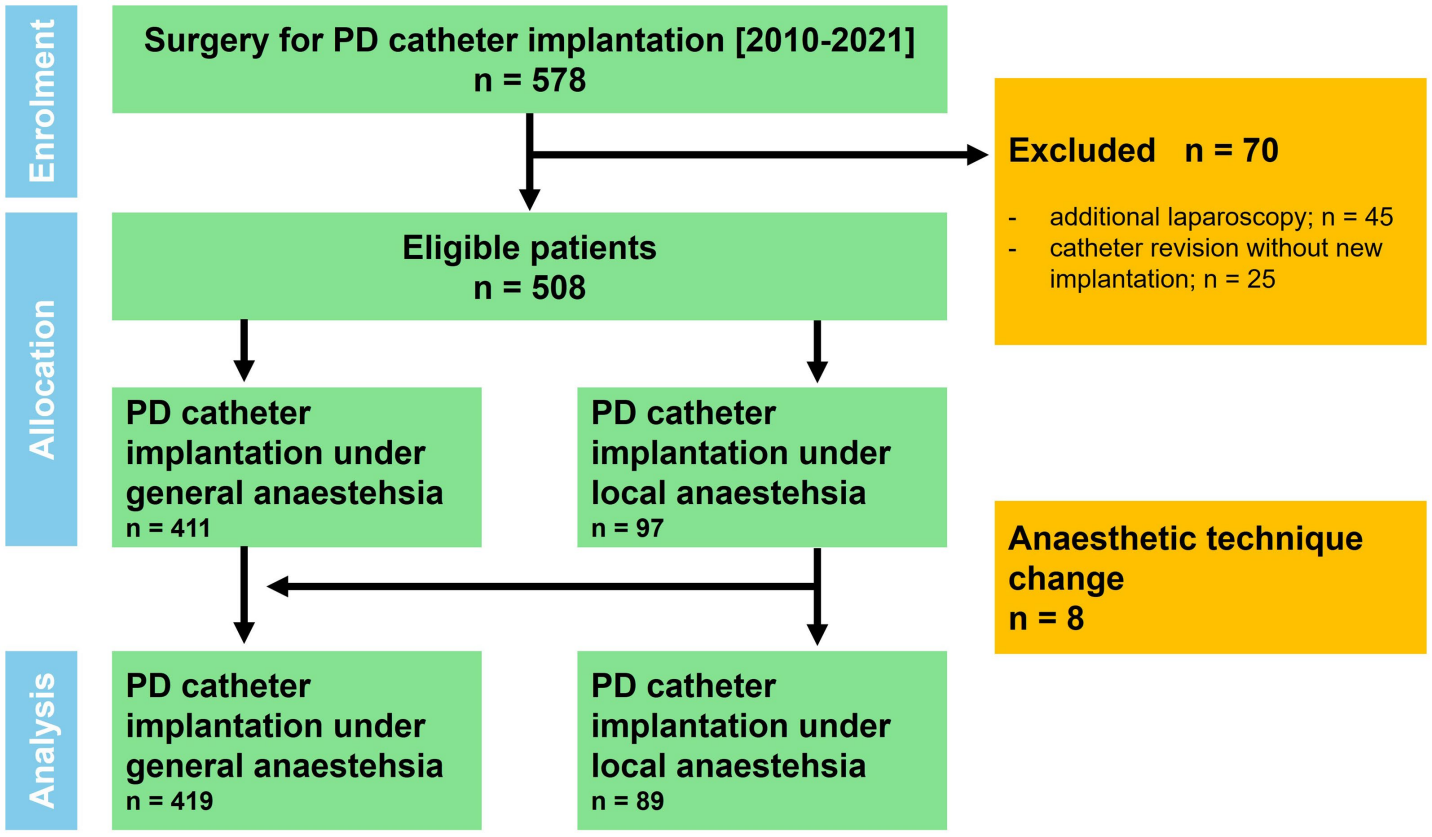
Patient flow chart.

This retrospective study included all consecutive surgical PD catheter insertions through mini-laparotomy performed between January 2010 and December 2021. To ensure that the study solely focused on intraoperative anesthesia during PD catheter placement, the following secondary exclusion criteria were established: additional use of laparoscopy (*n* = 45) or revision surgery without insertion of a new PD catheter (*n* = 25; Figure 1).

Patient data, including general patient characteristics, surgical characteristics such as need for additional procedures, postoperative surgical complications, and perioperative resource utilization including duration in the OR, recovery room and intensive care, were analyzed retrospectively from the prospectively maintained institutional database. Perioperative white blood cell count (WBC) and C-reactive protein (CRP) levels obtained from clinical routine laboratory examinations were used to assess the systemic inflammatory response to surgery. Accordingly, Preoperative values were defined as the last measurements obtained before surgery. Postoperatively, peak WBC and CRP values recorded up to postoperative day (POD) 4 were analyzed to capture the maximum inflammatory response attributable to surgical trauma. In addition, laboratory values at hospital discharge were evaluated to describe the postoperative inflammatory course.

### 2.2 Surgery and perioperative patient care

Peritoneal dialysis catheters were routinely implanted paramedian on the left side. A skin incision of approximately 5–7 cm was made in the left mid-abdomen, lateral to the midline and below the umbilicus. After dissection of the subcutaneous tissue, the anterior rectus sheath was incised, the rectus muscle was split longitudinally, and the peritoneum was opened over an area of approximately 1 cm^2^. Two purse-string sutures were placed at the peritoneum, and the catheter was advanced into the pelvic cavity under radiographic control. The catheter was then fixed to the peritoneum, guided cranially through the rectus sheath, and exteriorized laterally through the subcutaneous tissue and skin. After confirming adequate inflow and outflow, the anterior rectus sheath was closed, which was followed by layered wound closure. The majority of PD catheters were Oreopoulos-Zellermann types; in few cases, Tenckhoff double-cuff catheters were implanted at the specific request of referring outpatient nephrologists, who admitted the patients and subsequently continued their care. Postoperatively, the catheters were flushed daily by a nurse specialized in PD and typically first used for dialysis after approximately 14 days.

### 2.3 Statistical analyses

Statistical analyses were performed using GraphPad Prism (Version 10, GraphPad Software, San Diego, CA, USA). The patient cohort was divided into patients who underwent GA with endo-tracheal intubation or laryngeal mask (GA-group; *n* = 419) and in those who underwent local or regional anesthesia, respectively, with or without analgosedation (LA-group; *n* = 89). The latter group comprised clinically established techniques including local infiltration anesthesia and regional anesthesia (transversus abdominis plane block), with or without minimal to moderate analgosedation. These approaches were aggregated to reflect real-world perioperative practice. Patients who required an intraoperative procedural change from LA to GA (*n* = 8), were transferred to the GA group (Figure 1).

The LA and GA patient cohorts were subdivided according to the preoperative ASA score (ASA < 4 versus ASA ≥ 4) as well as according to isolation requirements in the OR area due to colonization with MDR organisms (Non-ISO versus ISO), in line with local policies and official guidelines (24, 25). Due to infection control regulations, postoperative monitoring in the post-anesthesia care unit (PACU) was avoided for patients in the ISO subgroups.

Categorical data of both groups were analyzed using Fisher’s exact or Pearson’s X2 test. Two-group comparisons of continuous variables were performed with two-tailed, unpaired Student’s T-test. One-way analysis of variance (ANOVA) was employed to assess global effects among continuous variables across multiple subgroups; where appropriate, Tukey’s post-hoc test was applied to determine pairwise differences while adjusting for multiple comparisons.

In boxplots, the central bar represents the median, whiskers indicate the 5th–95th percentile range, and individual points represent outliers. Boxes extend from the 25th to the 75th percentile, indicating the interquartile range, and the cross denotes the mean.

Data in tables were presented as mean ± standard deviation for continuous variables and as *n* (%) for categorical variables.

To address confounding by indication due to baseline differences between anesthesia groups, additional multivariable regression analyses were performed. Separate multivariable logistic regression models were fitted for perioperative safety outcomes, including intraoperative catecholamine therapy, postoperative intensive care, prolonged intensive care unit stay (>5 days), postoperative (re-) intubation, and 30-day mortality. Models included anesthesia type (local versus general), age, ASA classification, coronary artery disease, chronic pulmonary disease, isolation status, and extended surgical procedures. Model discrimination was assessed using the area under the receiver operating characteristic curve (AUC), and calibration was evaluated using the Hosmer–Lemeshow goodness-of-fit test.

As a sensitivity analysis for perioperative efficiency, a multivariable linear regression model was applied to total operating room time as a surrogate of perioperative resource utilization. Covariates included anesthesia type, age, ASA score, isolation status, and extended procedures.

A *p*-value ≤ 0.05 was considered statistically significant.

## 3 Results

### 3.1 General characteristics of the patient cohort

A total of 508 procedures met the final inclusion criteria and were included in the study: 419 PD catheters were implanted under GA (intubation: *n* = 357; laryngeal mask: *n* = 62) and 89 under local or regional anesthesia techniques (LA-group), i.e., local infiltration anesthesia (*n* = 75) or transversus abdominis plane block (*n* = 14) with (*n* = 87) or without (*n* = 2) additional analgesic sedation.

Patients who received LA were generally older and had a higher prevalence of chronic and critical illnesses, as reflected by significantly higher ASA scores. In the LA group, 49.4% of patients were classified as ASA score = 4, in comparison with 14.3% in the GA group. Conversely, the proportion of patients undergoing additional procedures beyond PD catheter insertion was higher in the GA cohort, most commonly involving synchronous hernia repairs. Despite these differences, the overall duration of surgery did not differ between the two groups (Table 1).

**Table 1:** Characteristics of patients, surgical procedures, and perioperative outcome.

Variable	Local anesthesia (*n* = 89)	General anesthesia (*n* = 419)	*p*-value
Patient characteristics			
Female gender [*n patients*]	26 (29.2%)	148 (35.3%)	0.3252
Age [*years*]	71.0 ± 13.3	61.6 ± 15.3	< 0.0001
Body mass index [*kg/m^2^*]	26.4 ± 5.5	26.8 ± 5.6	0.4856
ASA [*score*]	3.4 ± 0.6	3.0 ± 0.5	< 0.0001
1 [*n patients*]	-	-	
2 [*n patients*]	5 (5.6%)	52 (12.4%)	
3 [*n patients*]	40 (44.9%)	306 (73.0%)	
4 [*n patients*]	44 (49.4%)	60 (14.3%)	
5 [*n patients*]	-	1 (0.2%)	
Comorbidities			
Arterial hypertension [*n patients*]	80 (89.9%)	383 (91.4%)	0.6810
Coronary artery disease [*n patients*]	56 (62.9%)	149 (35.6%)	< 0.0001
Chronic pulmonal disease [*n patients*]	32 (36.0%)	101 (24.1%)	0.0242
Diabetes mellitus [*n patients*]	54 (60.7%)	256 (61.1%)	1
Chronic liver disease [*n patients*]	18 (20.2%)	50 (11.9%)	0.0575
Systemic immunosuppression [*n patients*]^#^	18 (20.2%)	71 (17.0%)	0.4460
Peripheral artery disease [*n patients*]	14 (15.7%)	55 (13.1%)	0.4987
Previous solid organ transplantation	6 (6.7%)	40 (9.6%)	0.5418
Previous malignoma [*n patients*]	18 (20.2%)	69 (16.5%)	0.4382
Active smoking [*n patients*]	9 (10.1%)	70 (16.7%)	0.1468
Active alcohol abuse [*n patients*]	3 (3.4%)	6 (1.4%)	0.1972
Previous abdominal surgery [*n patients*]	30 (33.7%)	168 (40.3%)	0.2831
Characteristics of surgical procedures			
Primary PD catheter placement [*n patients*]	83 (93.3%)	366 (87.4%)	0.1444
Catheter implantation left-sided [*n patients*]	78 (87.6%)	372 (88.8%)	0.7164
Right-sided [*n patients*]	11 (12.4%)	47 (11.2%)	
Duration of surgery [min]	44.3 ± 14.7	48.52 ± 26.3	0.1433
Extended procedures [*n patients*]	4 (4.5%)	66 (15.8%)	0.0036
Herniotomy [*n patients*]	3	56	
Previous catheter explantation [*n patients*]	1	10	
Perioperative outcome			
Intraoperative catecholamine therapy [*n patients*]	33 (37.1%)	106 (25.3%)	0.0265
Postoperative catecholamine therapy [*n patients*]	8 (9.0%)	21 (5.0%)	0.2038
Post-anesthesia care unit [*n patients*]	57 (64.0%)	344 (82.1%)	0.0002
Direct transfer to general ward [*n patients*]	13 (14.6%)	44 (10.5%)	0.2691
Direct transfer to intensive care unit [*n patients*]	19 (21.3%)	31 (7.4%)	0.0003
Postoperative intensive care [*n patients*]	39 (43.8%)	72 (17.2%)	<0.0001
Total postoperative duration at intensive care unit [d]	2.2 ± 5.2	1.1 ± 4.9	0.0709
Postoperative intensive care >5 days [*n patients*]	10 (11.2%)	20 (4.8%)	0.0260
Total postop hospital stay [d]	10.6 ± 7.9	8.6 ± 7.2	0.0222
Postoperative (re-) intubation [*n patients*]	6 (6.7%)	6 (1.4%)	0.0093
Seroma/hematoma [*n patients*]	10 (11.2%)	28 (6.7%)	0.1795
30 d mortality [*n patients*]	11 (12.4%)	15 (3.6%)	0.0021
Re-do surgery until postoperative day 90 [*n patients*]	6 (6.7%)	35 (8.4%)	0.8301
Onset of peritoneal dialysis [postoperative days]	10.4 ± 9.7	10.6 ± 10.6	0.8417

Data are given in mean ± standard deviations or *n* (%). ^#^including HIV, chemotherapy 6 weeks prior to surgery, or history of (solid) organ transplantation. ASA, American Society of Anesthesiologist’s classification of physical health (ASA) score.

**Figure 2:**
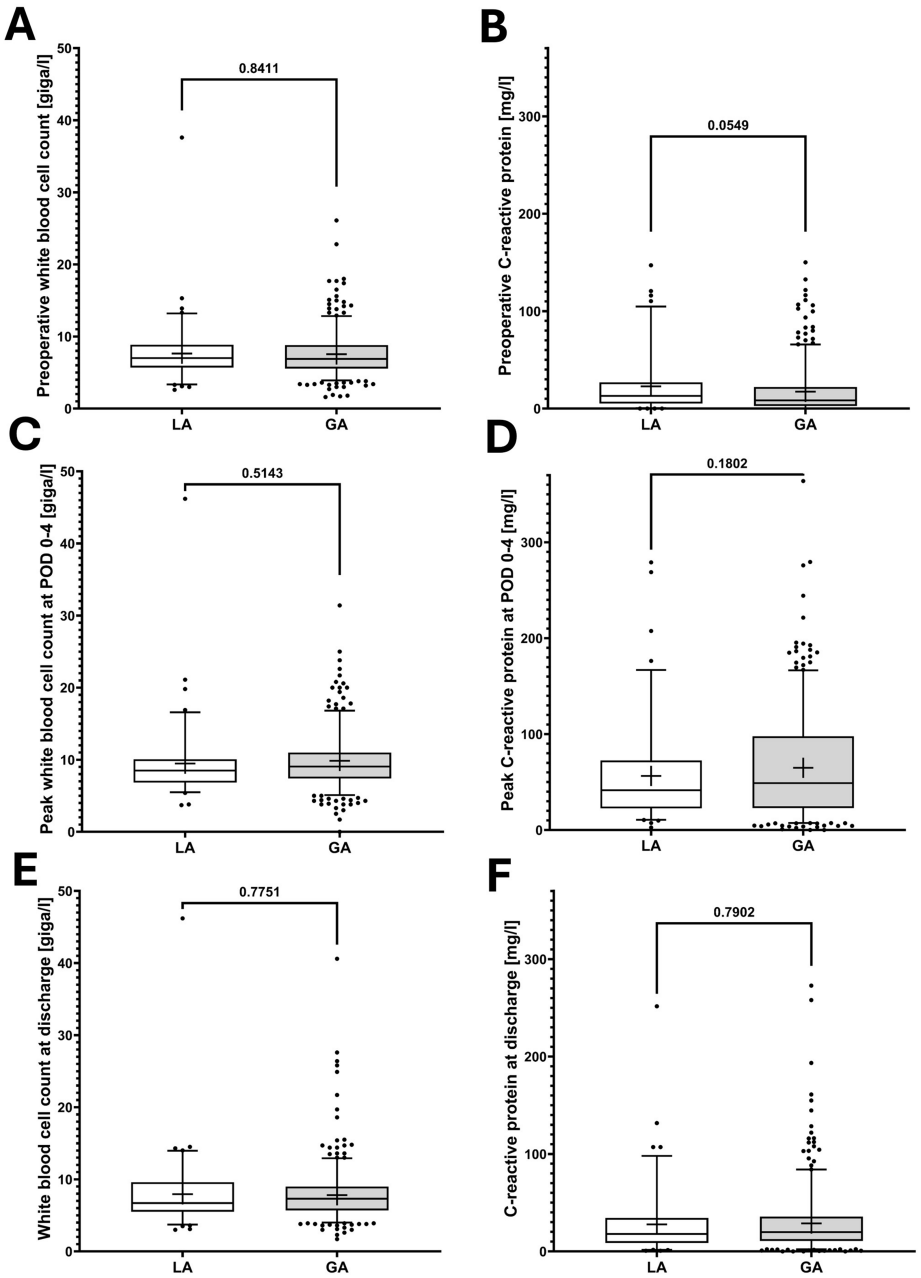
Perioperative systemic inflammation in patients who underwent local (LA) versus general anesthesia (GA) for peritoneal dialysis catheter implantation. **(A,C,E)** Perioperative white blood cell counts in peripheral blood. **(B,D,F)** perioperative C-reactive protein values in peripheral blood. *p*-values from Student T test indicate the significances of two-group comparisons. In boxplots, the central bar represents the median, whiskers indicate the 5th–95th percentile range, and individual points represent outliers. Boxes extend from the 25th to the 75th percentile, indicating the interquartile range, and the cross denotes the mean. POD, postoperative day.

Perioperative markers of systemic inflammation indicated equal surgical trauma in patients who underwent PD catheter implantation under LA versus GA (Figure 2).

Although patients who underwent PD catheter implantation under LA experienced higher rates of prolonged intensive care treatment and overall 30-day mortality, likely reflecting the greater overall illness severity in this subgroup, surgery-related complications as well the duration until onset of PD were comparable between the groups (Table 1).

### 3.2 Perioperative management depends on the type of anesthesia

**Figure 3:**
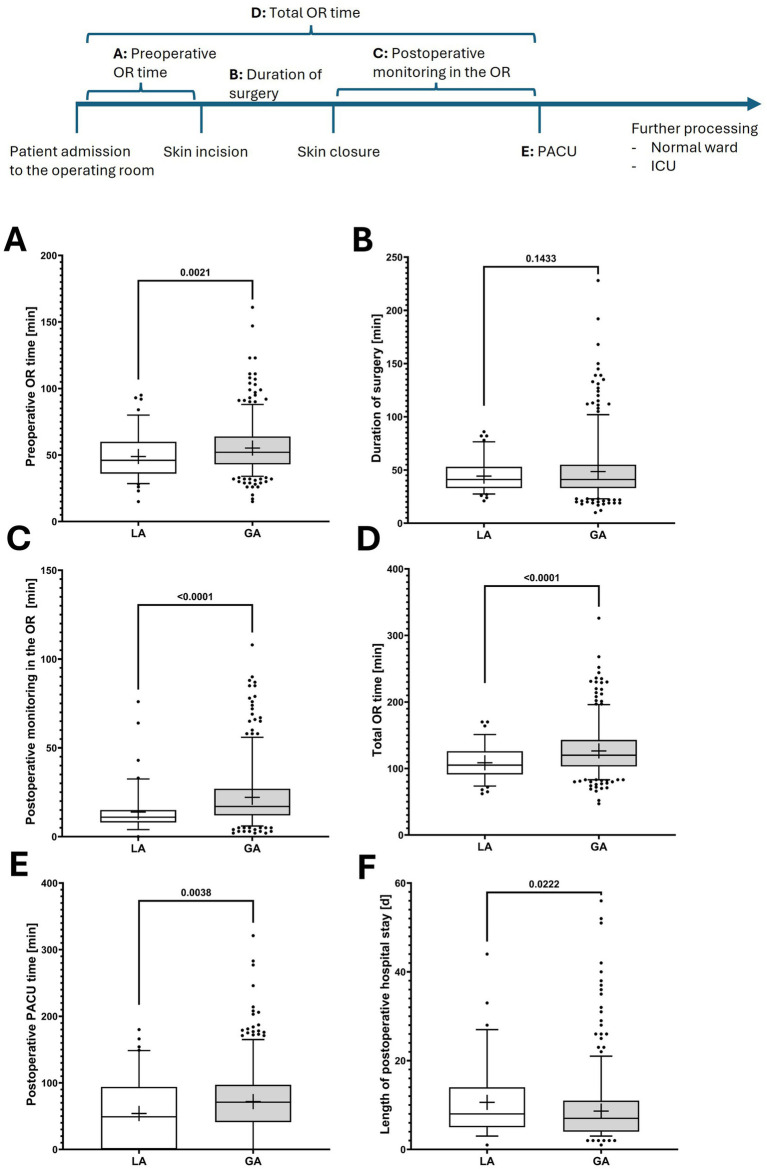
Perioperative management of patients who underwent local (LA) versus general anesthesia (GA) for peritoneal dialysis catheter implantation. The figure illustrates perioperative time intervals: **(A)** preoperative time in the operating room, **(B)** duration of surgery (skin incision to closure), **(C)** postoperative monitoring time in the operating room, **(D)** total time in the operating room (sum of preoperative, intraoperative, and postoperative monitoring periods), **(E)** postoperative stay in the post-anesthesia care unit (PACU), and **(F)** length of postoperative in-hospital stay. *p*-values from Student T test indicate the significances of two-group comparisons. In boxplots, the central bar represents the median, whiskers indicate the 5th–95th percentile range, and individual points represent outliers. Boxes extend from the 25th to the 75th percentile, indicating the interquartile range, and the cross denotes the mean. ICU, intensive care unit; OR, operating room; PACU, post-anesthesia care unit.

Despite baseline differences in patient comorbidities between the LA and GA groups, patients who underwent LA required significantly less time in the OR and had shorter stays in the PACU, indicating reduced postoperative monitoring. Consequently, patients in the LA-group were transferred more quickly to a definitive care unit, including the general ward or the intensive or the intermediate care unit (Figure 3).

Importantly, the increased need for postoperative intensive care and the prolonged stays in both the intensive care unit and the hospital observed in the LA group may reflect the greater prevalence and severity of comorbidities in comparison with those in the GA group.

### 3.3 Subgroup analysis of patients according to their comorbidities

In the subgroup of patients with ASA ≥ 4, the LA approach appeared to confer a clinical advantage, since the rate of intraoperative catecholamine therapy tended to be lower than that with the GA approach (LA: 47.7% versus GA: 67.2%; *p* = 0.0697). Although patients with ASA scores ≥4 were more frequently transferred directly to the intensive care unit than those with ASA scores <4, patients undergoing LA approaches were generally less often monitored in the PACU and more commonly transferred straight to the definitive ward, including the intensive care unit.

**Figure 4:**
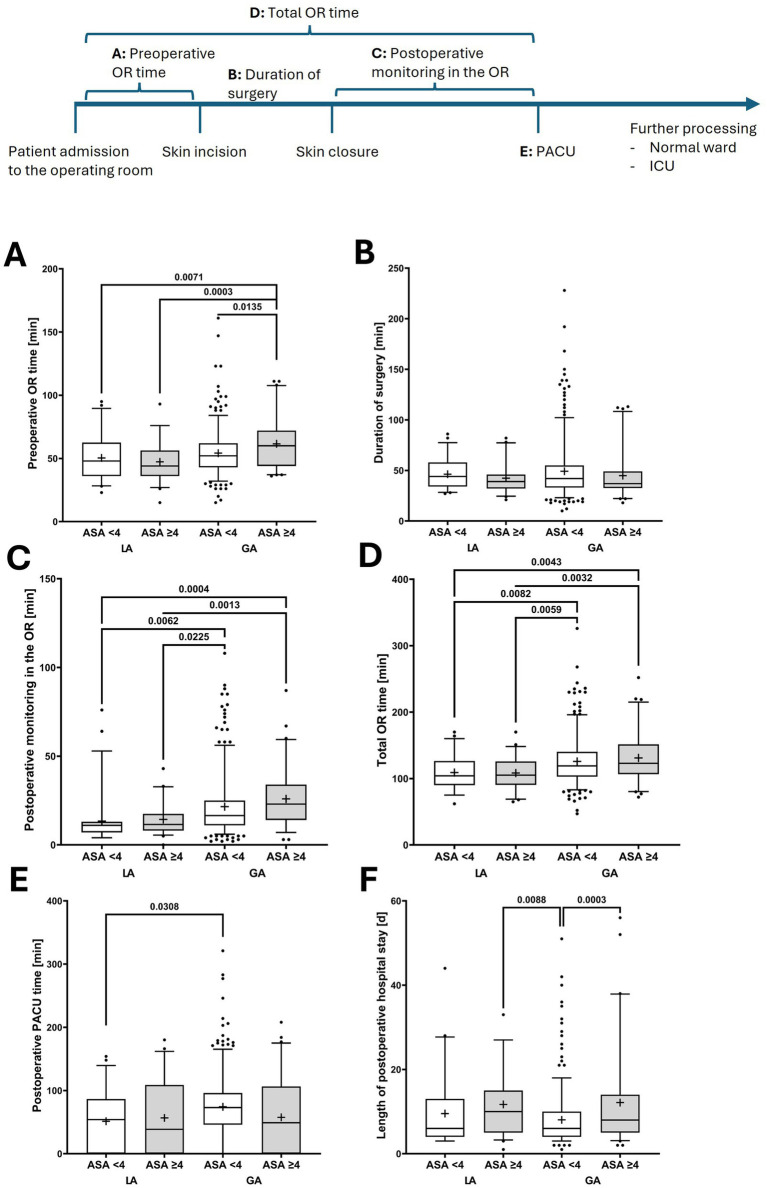
Perioperative management of patients stratified according to ASA score who underwent local (LA) versus general anesthesia (GA) for peritoneal dialysis catheter implantation. The figure illustrates perioperative time intervals: **(A)** preoperative time in the operating room, **(B)** duration of surgery (skin incision to closure), **(C)** postoperative monitoring time in the operating room, **(D)** total time in the operating room (sum of preoperative, intraoperative, and postoperative monitoring periods), **(E)** postoperative stay in the post-anesthesia care unit (PACU), and **(F)** length of postoperative in-hospital stay. ASA, American Society of Anesthesiologist’s classification of physical health (ASA) score. One-way ANOVA was employed to assess global effects across multiple subgroups; if applicable, Tukey’s post-hoc test was applied to determine pairwise differences while adjusting for multiple comparisons. *p*-values indicate the significances from Tukey’s post-hoc test. In boxplots, the central bar represents the median, whiskers indicate the 5th–95th percentile range, and individual points represent outliers. Boxes extend from the 25th to the 75th percentile, indicating the interquartile range, and the cross denotes the mean. ICU, intensive care unit; OR, operating room; PACU, post-anesthesia care unit.

From an economic perspective, patients with ASA scores ≥4 who underwent GA spent the longest time in the OR area. This was particularly evident for postoperative monitoring time in the OR, although this difference did not reach statistical significance in comparison with the findings for patients with ASA scores <4. In contrast, total duration in the OR area was markedly shorter in the LA group (Table 2; Figure 4).

**Table 2:** Perioperative management of patients stratified according to severity of comorbidities by ASA score: ASA < 4 versus ASA ≥ 4.

Variable	Local anesthesia (*n* = 89)		General anesthesia (*n* = 419)		*p*-value
	ASA < 4 (*n* = 45)	ASA ≥ 4 (*n* = 44)	ASA < 4 (*n* = 358)	ASA ≥ 4 (*n* = 61)	
Intraoperative catecholamine therapy [*n patients*]	12 (26.7%)	21 (47.7%)*	65 (18.2%)	41 (67.2%)^###^	<0.0001
Postoperative catecholamine therapy [*n patients*]	2 (4.4%)	6 (13.6%)	9 (2.5%)	12 (19.7%)^###^	<0.0001
Post-anesthesia care unit [*n patients*]	30 (66.7%)^§^	27 (61.4%)	304 (84.9%)^§^	40 (65.6%)^##^	<0.0001
Direct transfer to general ward [*n patients*]	10 (22.2%)	3 (6.8%)	40 (11.2%)	4 (6.6%)	0.0756
Direct transfer to intensive care unit [*n patients*]	5 (11.1%)^§^	14 (31.8%)*	14 (3.9%)^§^	17 (27.9%)^###^	<0.0001
Postoperative intensive care [*n patients*]	12 (26.7%)^§^	27 (61.4%)*	43 (12.0%)^§^	29 (47.5%)^###^	<0.0001
Postoperative intensive care >5 days [*n patients*]	3 (6.7%)	7 (15.9%)	14 (3.9%)	6 (9.8%)	0.0066

Data are given *n* (%). *Significantly different versus ASA <4 patients from the local anesthesia group (*p < 0.05; **p < 0.001; ***p < 0.0001). ^#^Significantly different versus ASA <4 patients from the general anesthesia group (^#^*p* < 0.05; ^##^*p* < 0.001; ^###^*p* < 0.0001). ^§^Significantly different between local versus general anesthesia patients. ASA, American Society of Anesthesiologist’s classification of physical health (ASA) score.

### 3.4 Subgroup analysis by isolation status due to colonization with multidrug-resistant organisms

Patients with chronic illnesses and frequent hospitalizations are at a high risk of carrying MDR bacteria. Consequently, the microbial status of surgical patients influences operative capacities, since MDR colonization necessitates isolation protocols within the OR area. This factor further amplifies the economic burden associated with different anesthesia strategies.

**Figure 5:**
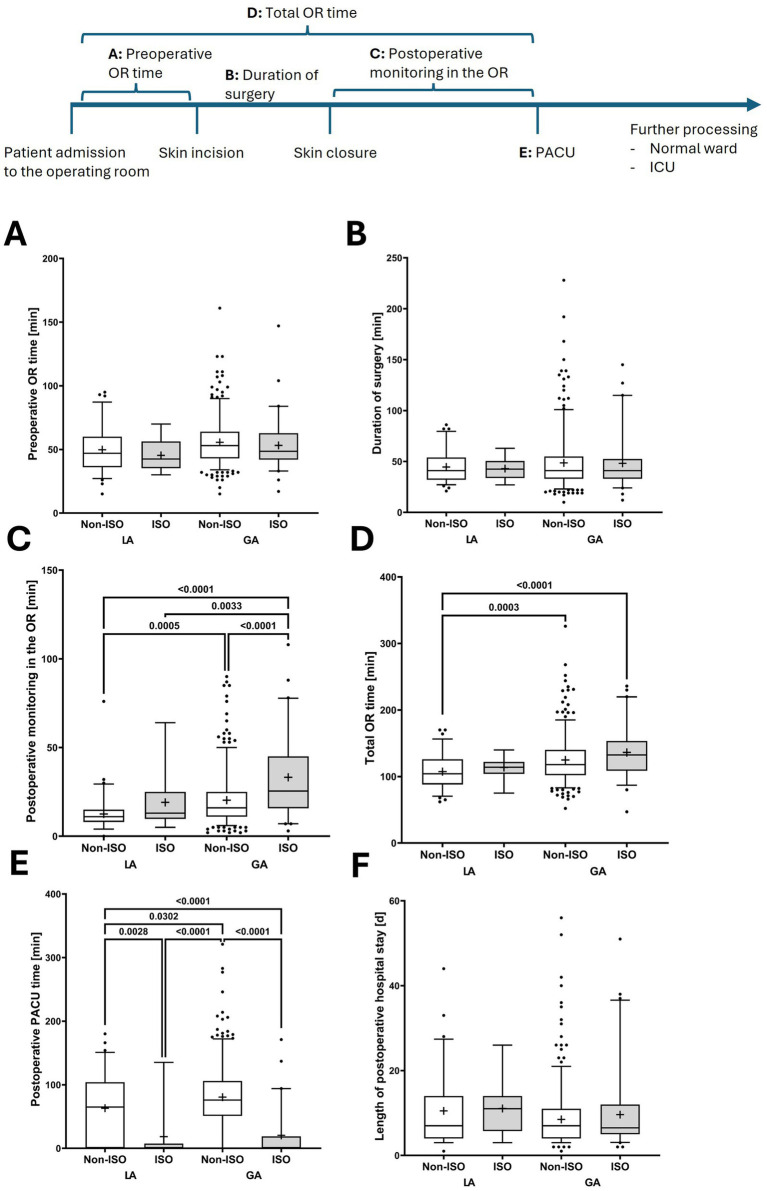
Perioperative management of patients stratified according to operating room isolation status due to colonialization with multidrug-resistant organisms (isolation required [ISO] vs. no isolation [Non-ISO]) undergoing peritoneal dialysis catheter implantation under local (LA) or general anesthesia (GA). The figure illustrates perioperative time intervals: **(A)** preoperative time in the operating room, **(B)** duration of surgery (skin incision to closure), **(C)** postoperative monitoring time in the operating room, **(D)** total time in the operating room (sum of preoperative, intraoperative, and postoperative monitoring periods), **(E)** postoperative stay in the post-anesthesia care unit (PACU), and **(F)** length of postoperative in-hospital stay. One-way ANOVA was employed to assess global effects across multiple subgroups; if applicable, Tukey’s post-hoc test was applied to determine pairwise differences while adjusting for multiple comparisons. *p*-values indicate the significances from Tukey’s post-hoc test. In boxplots, the central bar represents the median, whiskers indicate the 5th–95th percentile range, and individual points represent outliers. Boxes extend from the 25th to the 75th percentile, indicating the interquartile range, and the cross denotes the mean. ICU, intensive care unit; OR, operating room; PACU, post-anesthesia care unit.

While only a small number of patients with preoperatively detected MDR colonization underwent extended monitoring in the PACU, the ORs were disproportionately occupied by patients who underwent PD catheter implantation under GA, particularly those requiring isolation for postoperative monitoring. These patients had to be monitored in the OR area for extended periods, further limiting the efficiency of surgical units. Interestingly, patients receiving LA showed no significant differences in perioperative workflow in the OR, regardless of their MDR status and the need for isolation in the OR area (Table 3; Figure 5).

**Table 3:** Perioperative management of patients stratified according to operating room isolation status due to colonialization with multidrug-resistant organisms: patients requiring isolation (ISO) versus patients without isolation requirements (Non-ISO).

Variable	Local anesthesia (*n* = 89)		General anesthesia (*n* = 419)		*p*-value
	Non-ISO (*n* = 71)	ISO (*n* = 18)	Non-ISO (*n* = 359)	ISO (*n* = 60)	
Intraoperative catecholamine therapy [*n patients*]	27 (38.0%)^§^	6 (33.3%)	82 (22.8%)^§^	24 (40.0%)^#^	0.0045
Postoperative catecholamine therapy [*n patients*]	5 (7.0%)	3 (16.7%)	14 (3.9%)	7 (17.5%)^#^	0.0116
Post-anesthesia care unit [*n patients*]	53 (74.7%)^§^	4 (22.2%)***	329 (91.6%)^§^	15 (25.0%)^###^	<0.0001
Direct transfer to general ward [*n patients*]	5 (7.0%)	8 (44.4%)**	11 (3.1%)	33 (55.0%)^###^	<0.0001
Direct transfer to intensive care unit [*n patients*]	13 (18.3%)^§^	6 (33.3%)	19 (5.3%)^§^	12 (20.0%)^##^	<0.0001
Postoperative intensive care [*n patients*]	32 (45.1%)^§^	7 (38.9%)	56 (15.6%)^§^	16 (26.7%)^#^	<0.0001
Postoperative intensive care >5 days [*n patients*]	10 (14.1%)^§^	0	15 (4.2%)^§^	5 (8.3%)	0.0118

Data are given *n* (%). *Significantly different versus non-ISO patients from the local anesthesia group (*p < 0.05; **p < 0.001; ***p < 0.0001). ^#^Significantly different versus non-ISO patients from the general anesthesia group (^#^*p* < 0.05; ^##^*p* < 0.001; ^###^*p* < 0.0001). ^§^Significantly different between local versus general anesthesia patients.

### 3.5 Adjusted safety outcomes

In multivariable logistic regression analyses, perioperative safety outcomes were predominantly associated with baseline patient risk factors rather than anesthetic strategy. Higher ASA score emerged as the strongest and most consistent predictor across safety endpoints, being independently associated with intraoperative catecholamine therapy, postoperative intensive care unit admission, prolonged intensive care, and 30-day mortality. Chronic cardiopulmonary comorbidities were also independently associated with these outcomes.

After adjustment for these patient-related variables, anesthesia type was not independently associated with intraoperative catecholamine therapy, prolonged postoperative intensive care unit stay, postoperative (re-) intubation, or 30-day mortality (Table 4).

**Table 4:** Multivariable logistic regression analyses for postoperative safety outcomes.

Outcome	AUC *p* value Hypothesis	Events (n/N)	Adjusted odds ratio [95% confidence interval]; *p* value						
			LA vs GA	Age	ASA	CAD	Chronic pulmonary	Isolation status	Extended Procedures
Intraoperative catecholamine therapy	0.7894 *p* < 0.0001 pH-L: 0.0565	139/508	0.67 [0.37–1.17] *p* = 0.1607	1.03 [1.02–1.05] ***p* = 0.0002**	3.04 [1.94–5.86] ***p* < 0.0001**	2.29 [1.45–3.64] ***p* = 0.0004**	1.84 [1.14–2.95] ***p* = 0.0124**	1.48 [0.67–3.15] *p* = 0.3251	0.62 [0.29–1.26] *p* = 0.1917
Postoperative intensive care	0.7742 *p* < 0.0001 pH-L: 0.5764	111/508	1.98 [1.12–3.46] ***p* = 0.0183**	1.00 [0.98–1.02] *p* = 0.8559	4.07 [2.55–6.63] ***p* < 0.0001**	1.71 [1.03–2.83] ***p* = 0.0370**	1.778 [1.07–2.03] ***p* = 0.0256**	1.40 [0.62–3.01] *p* = 0.4118	0.79 [0.34–1.69] *p* = 0.5561
Postoperative intensive care >5 days	0.7193 *p* < 0.0001 pH-L: 0.1972	30/508	1.70 [0.68–4.04] *p* = 0.2486	0.9723 [0.95–1.0] ***p* = 0.00495**	2.69 [1.26–5.90] ***p* = 0.0098**	1.44 [0.61–3.42] *p* = 0.4062	2.38 [1.07–5.25] ***p* = 0.0330**	1.04 [0.23–3.32] *p* = 0.9486	0.67 [0.10–2.44] *p* = 0.5808
Postoperative (re-) intubation	0.7461 *p* = 0.0035 pH-L: 0.4479	12/508	3.39 [0.95–12.26] *p* = 0.0606	1.04 [0.99–1.10] *p* = 0.1658	0.76 [0.26–2.26] *p* = 0.6185	2.73 [0.74–12.36] *p* = 0.1343	0.69 [0.15–2.42] *p* = 0.5767	1.27 [0.07–7.40] *p* = 0.8293	0.74 [0.04–4.26] *p* = 0.7704
30 day mortality	0.8087 *p* < 0.0001 pH-L: 0.8918	26/508	2.02 [0.80–4.94] *p* = 0.1328	1.06 [1.02–1.11] ***p* = 0.0011**	2.43 [1.12–5.50] ***p* = 0.0253**	0.41 [0.17–0.99] ***p* = 0.0460**	1.75 [0.75–4.04] *p* = 0.1948	0.86 [0.13–3.36] *p* = 0.8509	0.35 [0.12–1.82] *p* = 0.2495

Separate multivariable logistic regression models were fitted for each outcome. Models included anesthesia type (local, LA versus general anesthesia, GA), age, American Society of Anesthesiologist’s classification of physical health (ASA) score, coronary artery disease (CAD), chronic pulmonary disease, isolation status, and extended surgical procedures. Results are presented as adjusted odds ratios with 95% confidence intervals and *p* values. Model discrimination was assessed using the area under the receiver operating characteristic curve (AUC), and calibration was evaluated using the Hosmer–Lemeshow (H-L) goodness-of-fit test. Bold values indicate significances.

### 3.6 Adjusted efficiency analysis

In multivariable linear regression analysis, local anesthesia remained independently associated with shorter total operating room time (adjusted *β* = −13.6 min; *p* = 0.0002). Isolation requirements and extended surgical procedures were the strongest independent drivers of prolonged operating room occupancy (Table 5).

**Table 5:** Multivariable linear regression analyses for perioperative operating room efficiency.

Outcome	Predictor	Adjusted *β* [min]	95% confidence interval	*p* value
Total operating room time [min]	Local versus general anesthesia	−13.56	−20.73 – −6.38	0.0002
	Age	−0.05	−0.23 – 0.13	0.6101
	ASA	+2.86	−2.19 – 7.92	0.2660
	Isolation status	+13.71	4.22–23.21	0.0047
	Extended procedure	+42.05	34.58–49.51	<0.0001

Multivariable linear regression models were applied to assess factors associated with perioperative total operating room time. Models included anesthesia type (local versus general), age, American Society of Anesthesiologist’s classification of physical health (ASA) score, isolation status, and extended procedures. Results are presented as adjusted regression coefficients (*β*) in minutes with 95% confidence intervals.

## 4 Discussion

This retrospective cohort study of 508 consecutive PD catheter insertions provided comprehensive data indicating that LA-based approaches are a viable and potentially advantageous alternative to GA during open surgical PD catheter placement in selected patients. Our findings demonstrate that despite being preferentially utilized in older, more comorbid patients with significantly higher ASA scores, LA achieved comparable surgical success rates while offering distinct perioperative and organizational advantages.

### 4.1 Clinical safety and general outcomes

The comparable surgical complication rates between the LA and GA groups, despite the significantly higher baseline morbidity in the LA cohort, supports the safety profile of LA techniques for PD catheter insertion. This finding aligns with the results of previous studies demonstrating the feasibility of LA for abdominal procedures in these high-risk patients (9–14). The high procedural success rate in our LA group, with only a small number of conversions to GA (*n* = 8), further corroborates these observations and compares favorably with published series reporting similar low conversion rates (12, 13).

Unadjusted analyses revealed a higher 30-day mortality in the LA group. Given the substantially greater baseline illness severity in these patients, particularly with respect to cardiopulmonary comorbidities, this finding is best interpreted in the context of patient selection rather than as an indicator of procedure- or anesthesia-related risk (15). Comparable postoperative inflammatory response markers (CRP and WBC) between groups further suggest that the anesthetic technique does not influence the degree of surgical trauma or postoperative inflammatory response.

The adjusted analyses provide a more differentiated understanding of these outcome patterns. Across all multivariable models, perioperative morbidity and mortality were predominantly driven by patient-related risk factors rather than by anesthetic strategy. Higher ASA scores and cardiopulmonary comorbidities emerged as the most consistent and clinically relevant predictors of adverse postoperative outcomes, including intensive care utilization and mortality. In addition, although analgosedation may theoretically influence perioperative outcomes in multimorbid patients, its potential impact cannot be disentangled from baseline patient risk in this retrospective cohort; importantly, the multivariable analyses consistently identified patient-related factors rather than anesthetic strategy as the primary drivers of intensive care utilization and other safety endpoints.

Although unadjusted 30-day mortality was higher in the LA group, this association did not persist after adjustment for baseline disease severity. In multivariable analyses, anesthesia type was not independently associated with intraoperative catecholamine requirement, prolonged intensive care treatment, postoperative (re-) intubation, or 30-day mortality. The independent association between local anesthesia and postoperative intensive care unit admission is therefore best interpreted as reflecting structured clinical decision-making and planned postoperative monitoring in multimorbid high-risk patients rather than an adverse effect of the anesthetic technique itself.

### 4.2 Efficiency and resource utilization

Our analysis revealed the substantial advantages of LA with respect to perioperative workflow efficiency. Patients undergoing LA required substantially less total OR time, primarily due to reduced preoperative preparation and postoperative monitoring requirements. These findings have relevant implications for OR capacity utilization, particularly in resource-constrained healthcare settings. Thereby it has previously shown, that ORs generate the highest contribution margins in hospital settings (17, 18). The reduced need for PACU resources in patients undergoing LA (64.0% versus 82.1%) represents improved patient flow. PACU bottlenecks are a well-recognized cause of OR delays and reduced throughput (20, 21, 26). By enabling more rapid transfer to definitive care units, LA may contribute to improved OR turnover and capacity utilization.

### 4.3 Management of high-risk patients

The subgroup analysis of patients with ASA score ≥4 patients yielded particularly compelling evidence for the benefits of LA in this critically ill population. The trend toward a reduced requirement for intraoperative catecholamine support in patients undergoing LA (47.7% in the ASA ≥ 4 subgroup of the LA group versus 67.2% in the ASA ≥ 4 subgroup of the GA group) indicates improved hemodynamic stability during surgery, likely reflecting the reduced cardiovascular stress associated with avoiding GA and positive pressure ventilation (27, 28). This finding is particularly relevant since hemodynamic instability is a major contributor to perioperative morbidity in patients with kidney failure (29, 30).

While postoperative monitoring requirements remained elevated in high-risk multimorbid patients regardless of anesthetic technique, the improved intraoperative stability suggests that LA may provide clinical benefits during the immediate perioperative period. This is consistent with literature demonstrating reduced cardiovascular complications with regional anesthesia in comparison with GA in high-risk populations (27, 31, 32).

### 4.4 Isolation protocols and perioperative resource utilization

Our analysis of patients requiring MDR isolation protocols highlighted a previously underappreciated benefit of LA in contemporary perioperative management. In this subgroup, the disproportionate OR occupancy associated with GA, which was driven by extended postoperative monitoring under isolation conditions, represented a major operational inefficiency. With the growing prevalence of MDR organisms in hospitalized populations, this observation shows increased relevance for healthcare systems (33–36). Importantly, the ability to perform PD catheter insertion under LA without compromising clinical outcomes while maintaining full isolation measures throughout the perioperative period offers substantial organizational advantages. This is particularly valuable given the resource-intensive nature of isolation procedures and their effects on OR scheduling flexibility.

### 4.5 Economic implications

Although this study did not include a formal cost-effectiveness analysis, the reduced perioperative resource utilization in the OR area associated with LA suggests potential organizational and economic relevance, which requires confirmation in dedicated cost-effectiveness analyses. A shorter total OR occupancy, reduced PACU utilization, and more efficient workflow all may contribute to lower direct costs per procedure. While the higher rate of postoperative intensive care unit admissions observed in patients undergoing LA likely reflects their greater baseline illness severity, including more advanced cardiopulmonary comorbidities rather than perioperative complications, it may partially offset some of these savings in this high-risk cohort.

Nevertheless, our data support the safety of surgical PD catheter insertions under LA, particularly in patients with high morbidity, while demonstrating substantial perioperative resource savings within the OR environment. Additional prospective studies incorporating comprehensive analyses of costs, including medication costs, personnel time, and overall hospital resource utilization, are required to provide robust quantitative data regarding the economic impact of anesthetic choice in surgical PD catheter placement independent of baseline patient characteristics.

### 4.6 Limitations and future directions

Several limitations of this retrospective analysis warrant discussion. The non-randomized design introduced selection bias, with sicker patients more likely to receive LA. While this reflects real-world clinical practice, it complicates direct outcome comparisons between anesthetic approaches. The single-center design may have limited the generalizability of the findings, particularly regarding institutional protocols and patient populations. Although the study spans a long inclusion period, surgical technique and perioperative management of PD catheter placement at our institution remained largely unchanged during the study period, limiting the potential impact of temporal practice evolution on the observed results. Nevertheless, subtle changes in perioperative processes over time cannot be fully excluded due to the retrospective design. Furthermore, patient-reported outcome measures such as pain scores satisfaction and need for rescue analgesia were not retrospectively available, representing a major limitation with respect to assessment of patient experience.

The local anesthesia group encompassed heterogeneous techniques, including local infiltration and regional blocks with or without analgosedation; although this reflects routine perioperative practice, technique-specific effects could not be analyzed separately due to limited subgroup sizes and should be addressed in future prospective studies.

In addition, no formal cost or cost-effectiveness analysis was performed; therefore, conclusions are limited to perioperative resource utilization and workflow efficiency rather than direct economic outcomes.

Despite these limitations, our data support the safety and perioperative efficiency of LA for surgical PD catheter insertion, especially in high-risk patients and those requiring isolation precautions. Future research should build on these findings through prospective randomized trials comparing anesthetic approaches in PD catheter placement. Such studies should incorporate detailed cost-effectiveness analyses, patient-reported outcome measures, and long-term catheter function assessments. The development of standardized protocols for anesthetic selection based on patient comorbidities and institutional factors may further optimize perioperative management. In addition, integration of LA into enhanced recovery after surgery pathways may represent a promising strategy to improve outcomes and resource utilization in this setting.

## 5 Conclusion

In comparison with GA, LA for open surgical PD catheter placement demonstrated perioperative safety while offering substantial advantages in perioperative efficiency and resource utilization within the OR environment. These benefits were particularly pronounced in high-risk multimorbid patients and those requiring isolation precautions, populations that constitute an increasing proportion of patients with end-stage renal disease. Incorporation of LA into surgical PD catheter placement protocols provides a practical strategy to enhance patient care and addresses modern efficiency requirements in healthcare systems.

## Data Availability

The raw data supporting the conclusions of this article will be made available by the authors, without undue reservation.
